# Tumor purity as a prognosis and immunotherapy relevant feature in cervical cancer

**DOI:** 10.18632/aging.203714

**Published:** 2021-11-29

**Authors:** Yali Deng, Zewen Song, Li Huang, Zhenni Guo, Binghua Tong, Meiqing Sun, Jin Zhao, Huina Zhang, Zhen Zhang, Guoyin Li

**Affiliations:** 1 The Second Xiangya Hospital, Central South University, Changsha, Hunan, China; 2 Department of Oncology, The Third Xiangya Hospital, Central South University, Changsha, Hunan, China; 3 College of Life Science and Agronomy, Zhoukou Normal University, Zhoukou, Henan, China; 4 Academy of Medical Science, Zhengzhou University, Zhengzhou, Henan, China

**Keywords:** gynecologic cancer, tumor purity, B cell infiltration, immunity, prognosis

## Abstract

Background: Tumor purity plays a vital role in the biological process of solid tumors, but its function in gynecologic cancers remains unclear. This study explored the correlation between tumor purity and immune function of gynecological cancers and its reliability as a prognostic indicator of immunotherapy.

Methods: Gynecological cancer-related datasets were downloaded from The Cancer Genome Atlas (TCGA). Tumor purity was calculated by the ESTIMATE algorithm. A LASSO Cox regression analysis was performed to construct the risk score model. A Kaplan–Meier Plotter was used to explore the relationships between tumor purity and cancer prognosis. We performed the Kyoto Encyclopedia of Genes and Genomes (KEGG) and Gene Set Enrichment Analysis (GSEA) to explore the pathways in the subgroups. A nomogram was used to quantitatively assess the cancer prognosis.

Results: Tumor purity was negatively correlated with B cell infiltration in cervical squamous cell carcinoma and endocervical adenocarcinoma (CESC). Approximately 420 genes were positively associated with B cell infiltration and CESC prognosis and were enriched in immune-related signaling pathways. There were 11 key genes used to construct a risk score model. The low-risk group had a higher immune score and better prognosis than the high-risk group. A nomogram based on risk score, T stage, and clinical-stage had good predictive value in quantitatively evaluating CESC prognosis.

Conclusions: This study is the first to reveal the correlation between tumor purity and immunity in CESC and suggests that low-risk patients may be more sensitive to immunotherapy. This provides a theoretical basis for the clinical treatment of CESC.

## INTRODUCTION

Gynecological cancer is a serious threat to women’s health worldwide, especially cervical squamous cell carcinoma and endocervical adenocarcinoma (CESC), ovarian cancer (OV), uterine corpus endometrial carcinoma (UCEC), and uterine carcinosarcoma (UCS). Globally, CESC is the fourth most common female malignancy and the second-highest cause of female cancer-related deaths [1, 2]. Human papillomavirus infection (HPV) is the main cause of cervical cancer. Cervical cancer will be a preventable disease with increased HPV screening and vaccines. In recent years, the survival rate of cervical cancer patients has significantly improved, with the 5-year survival rate increasing to 68%, but the median survival time of patients with advanced cervical cancer is only 16.8 months [3]. TNM staging is vital for clinicians to make CESC treatment plans. Unfortunately, cases with the same TNM stage may have vastly different clinical outcomes. Therefore, it is urgent to identify the key factors that can accurately predict CESC prognosis.

Previous studies have shown that tumor purity is a potential prognostic tumor indicator [4, 5]. However, at present, there are very few studies on tumor purity in gynecological cancer. Tumor purity refers to the proportion of tumor cells in the tumor microenvironment (TME). TME includes a variety of cell populations, such as stromal cells, fibroblasts, endothelial cells, and immune cells, which play key roles in tumor occurrence and development [6]. The cells and molecular components in TME may affect therapy outcomes [6]. However, the role of tumor purity in cervical cancer remains unclear and requires further research. In the TME, immune and stroma cells are the main components of normal cells and exert important biological roles in tumor processes [7]. In 2013, Yoshihara et al. proposed the ESTIMATE algorithm to calculate the immune and stromal score, which represents the level of immune cell infiltration in the tumor [7].

Immune cell infiltration is closely related to the clinical therapeutic effect in various tumors [8]. Immune cells, including congenital and adaptive immune cell populations, such as dendritic cells (DCs), macrophages, neutrophils, T cells, and B cells, are involved in active and suppressive immune functions [9]. Existing studies have confirmed that more T cells are associated with a better prognosis in cervical cancer patients [10, 11]. M1 macrophages play an anti-cancer role in some carcinomas, while M2 macrophages play a pro-cancer role [12]. Neutrophils can promote tumor occurrence and development through complex mechanisms. Therefore, more neutrophils are related to a worse prognosis in patients [13]. Reports on the prognostic value of CD20+ B cells on carcinomas are contradictory, and further research is needed [14].

In this study, we calculated the tumor purity of CESC, OV, UCEC, and UCS with the ESTIMATE algorithm and found that only CESC prognosis was related to tumor purity. A Kaplan–Meier analysis showed that patients with low tumor purity had a better prognosis. GSEA suggested that genes in the low purity subgroup were enriched in immune-related signaling pathways. The level of B cells infiltration was negatively correlated with tumor purity in CESC. The B cell-related risk score was constructed by 11 key genes identified by LASSO regression analysis. A COX regression analysis was performed to screen the independent CESC prognostic factors, which were used to construct the nomogram to quantitatively evaluate CESC prognosis. Our study is the first to reveal the relationship between B cells infiltration and CESC tumor purity of CESC and to construct a reliable, clinically relevant prognostic model.

## MATERIALS AND METHODS

### Data acquisition and processing

The normalized expression and clinical data of four gynecologic cancers, including cervical squamous cell carcinoma and endocervical adenocarcinoma (CESC), ovarian serous cystadenocarcinoma (OV), uterine corpus endometrial carcinoma (UCEC), and uterine carcinosarcoma (UCS), were acquired from the GDC hub of the UCSC Xena website (http://xena.ucsc.edu/public) and were processed as reported in our previous work [15].

### Tumor purity and immune infiltration

Immune, stromal, and ESTIMATE scores were calculated by the ESTIMATE package in R software-based on Yoshihara et al. [7]. The empirical cumulative distribution function of the marker genes and the remaining genes was calculated according to the sample gene expression values. The integration of the differences between the empirical cumulative distribution functions was used to calculate the statistic based on absolute expression rather than differential expression. The tumor purity was estimated by the following formula: Tumor_purity = cos (0.6049872018 + 0.0001467884*ESTIMATEScore). The infiltration of immune cells was evaluated by the ssGSEA and TIMER algorithms, as reported in our previous study [16, 17].

### Functional and enrichment analyses

Kyoto Encyclopedia of Genes and Genomes (KEGG) enrichment analyses were performed using the ClueGo plug-in in the Cytoscape software. Gene set enrichment analysis (GSEA) was performed to explore the difference in pathways between the high and low tumor purity subgroups. The pathways were considered significantly enriched when the following criteria were met: nominal p-value < 0.05, false discovery rate q-value < 0.25, and absolute normalized enrichment score > 1.

### Development of the prognostic model

A least absolute shrinkage and selection operator (LASSO) Cox regression analysis was performed using the glmnet package in R. The 11 key genes generated by LASSO and their correlation coefficients were used to estimate the new score as follows: Score = -0.07761*C16orf54 - 0.04204*CHIT1 - 0.16457*DPEP2 - 0.13164*GNG8 - 0.99003*GTSF1L - 0.13794*IKZF3 - 0.42018*LILRA4 - 0.05666*POU2AF1 - 0.06302*S1PR4 - 0.74311*TRAV34 - 0.13761*ZBTB32. The risk score was further calculated by subtracting the minimum from the score and dividing by the absolute value of the maximum, as follows: risk score = (Score - min(Score)) / abs(max(Score)).

### Nomogram development and evaluation

The univariate and multivariate COX regression analyses were performed using survival and survminer packages in R to identify independent risk factors for CESC patients. The nomogram was constructed using rms package in R (Version 5.1-3.1, https://cran.r-project.org/web/packages/rms/) based on the independent risk factors. The consistency of actual outcome frequency and model prediction probability was evaluated by the concordance index (C-index).

### Statistical analysis

The data were analyzed by R software (version 4.0.2). Packages in R used for data analysis and graph plotting included estimate, glmnet, ggplot2, GSVA, limma, survminer, survival, tidyverse, dplyr, and plyr. The median value of tumor purity or risk score was used as the cut-off value for the two subgroups. A value of *p* < 0.05 was considered statistically significant (*, *p* < 0.05; **, *p* < 0.01; ***, *p* < 0.001; ****, *p* < 0.0001).

### Ethical approval and consent to participate

The data sets involved in this study were downloaded from public databases and did not require ethical approval.

### Availability of data and material

All data analyzed during this study are available in public databases.

## RESULTS

### Associations of tumor purity with prognosis and clinical features

**Figure 1:**
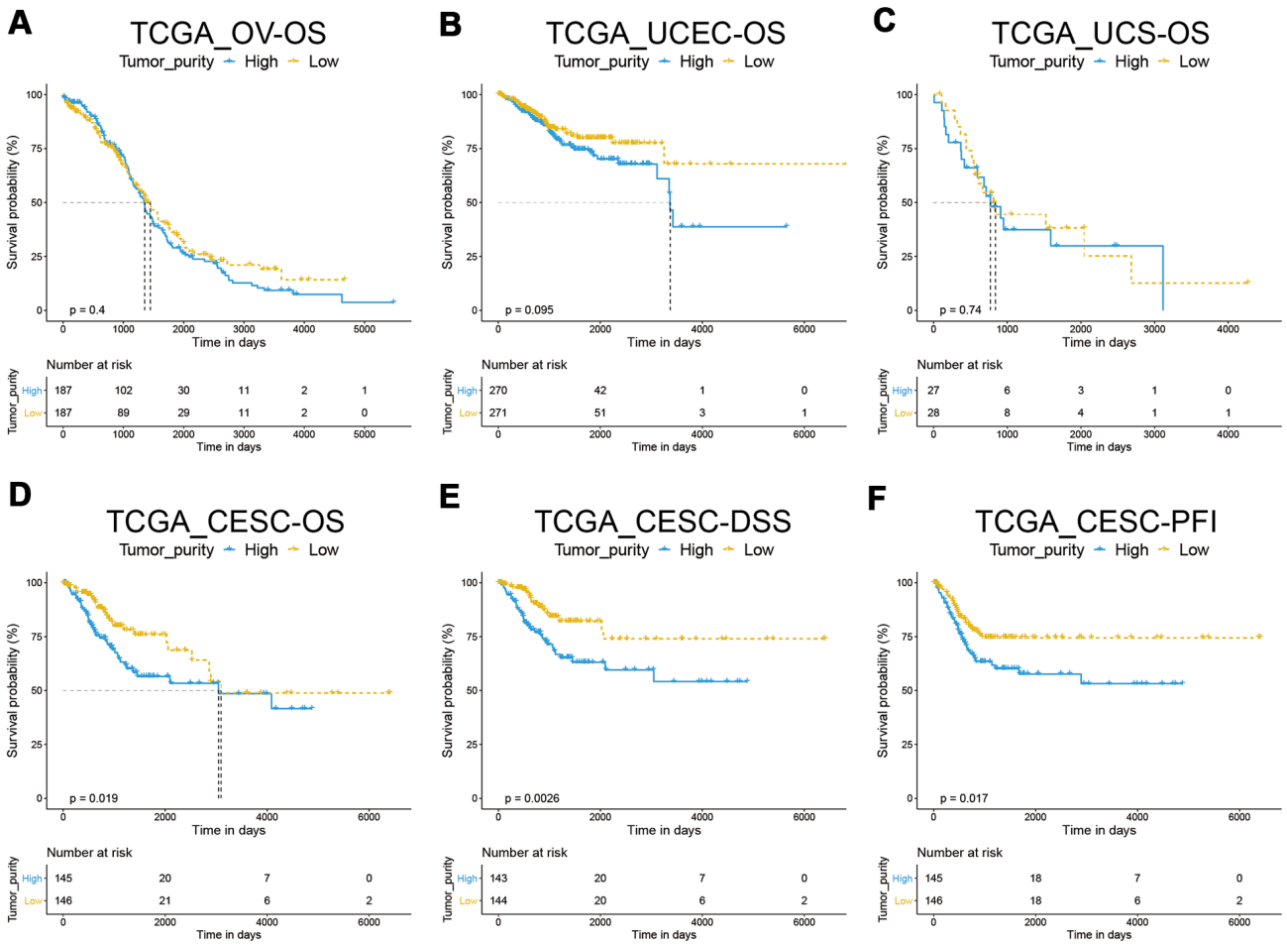
**Correlation between tumor purity and gynecological cancer prognosis.** (**A**–**C**) A Kaplan–Meier survival analysis indicated that tumor purity was not associated with OV, UCEC, and UCS overall survival. (**D**–**F**) A Kaplan–Meier survival analysis indicated that the low tumor purity subgroup of CESC had a better prognosis.

We analyzed a total of 291 cervical squamous cell carcinoma and endocervical adenocarcinomas (CESC), 542 uterine corpus endometrial carcinomas (UCEC), 376 ovarian serous cystadenocarcinomas (OV), and 55 uterine carcinosarcomas (UCS). We used the ESTIMATE algorithm to assess the tumor purity of the four carcinomas (Supplementary Table 1). Based on the median value, the patients were divided into high and low purity subgroups, respectively. Kaplan–Meier analysis suggested that there was no significant correlation between tumor purity and overall survival (OS) of patients with UCEC, OV, and UCS (Figure 1A–1C)]. CESE patients with low tumor purity showed longer OS (*p* = 0.019, Figure 1D). Moreover, the low tumor purity population from the TCGA_CESC dataset demonstrated a significantly longer disease-free survival (DSS, p = 0.0026, Figure 1E) and progression-free interval (PFI, *p* = 0.017, Figure 1F) than those with high tumor purity. We then analyzed the impact of tumor purity on the clinical characteristics of patients with CESC and found no significant difference in tumor purity based on histological grades, TNM stages, clinical stages, or age. (Supplementary Figure 1A–1F).

### Low tumor purity represents a stronger immune phenotype

**Figure 2:**
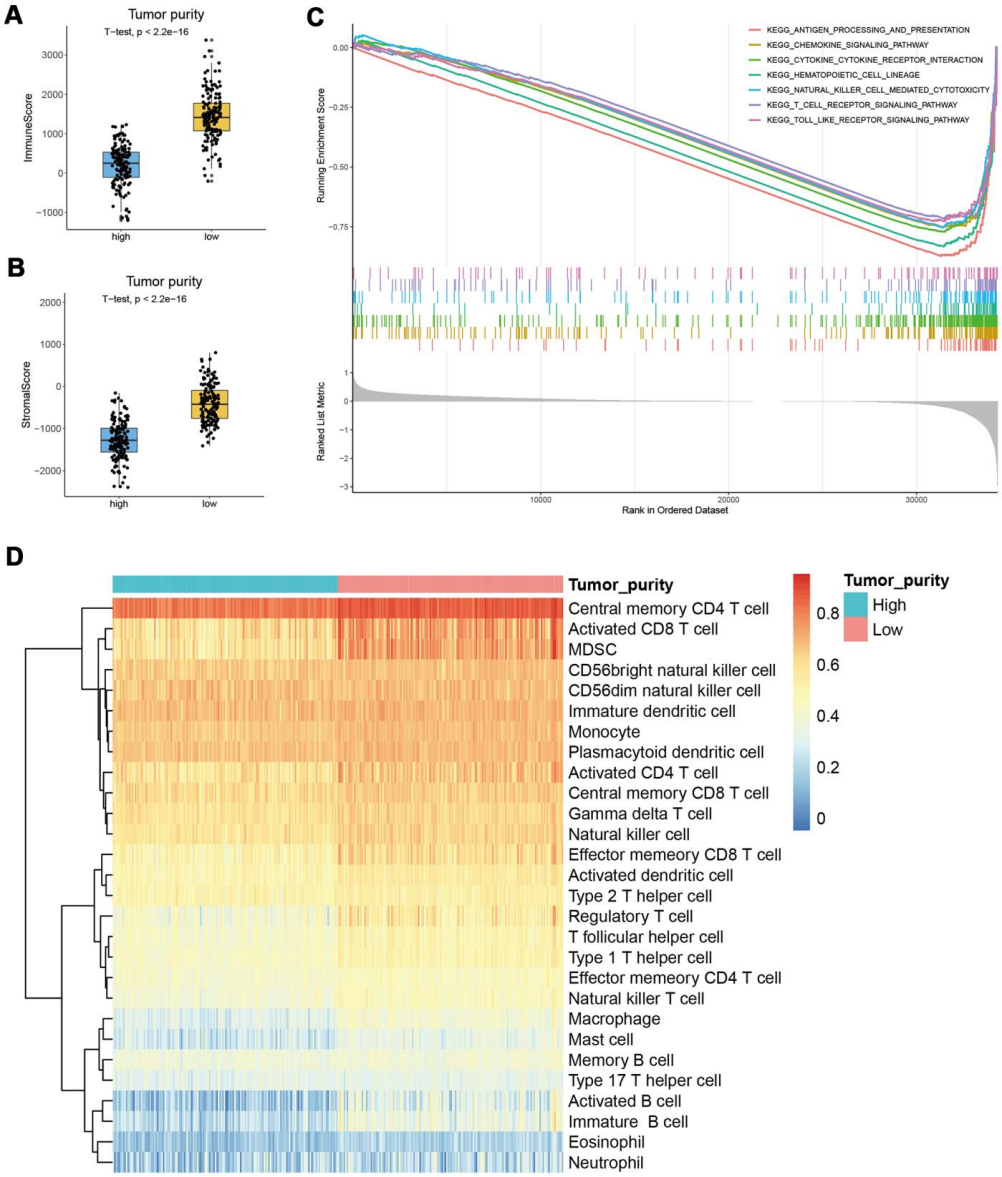
**Correlation between tumor purity and immunity in CESC.** (**A**, **B**) The immune and stromal scores of the low tumor purity subgroup were significantly higher than the high tumor purity subgroup. (**C**) GSEA results suggested that genes in the low tumor purity subgroup were mainly enriched in immune-related signaling pathways. (**D**) ssGSEA results showed that most immune cells were significantly increased in the low tumor purity subgroup.

In the tumor microenvironment (TME), immune and stromal cells are two major non-tumor components related to tumor prognosis [18, 19]. To assess the relationship between tumor purity and immunity, we analyzed the immune and stromal scores by the ESTIMATE algorithm and found that the scores of low tumor purity samples were significantly higher than those with high tumor purity, suggesting that the proportion of non-tumor cells in the low tumor purity group was higher (Figure 2A, 2B). To further explore the different molecular mechanisms between the two subgroups, we performed GSEA in the TCGA_CESC data set. The results suggested that genes in the low tumor purity subgroup were mainly enriched in the immune-related signaling pathways, such as antigen processing and presentation, chemokine signaling pathway, cytokine-cytokine receptor interaction, etc. (Figure 2C). We then analyzed and compared by ssGSEA the infiltration level of the immune cells between the two subgroups and found that most immune cells, such as MDSC and activated CD8 T cells, were significantly increased in the low tumor purity subgroup (Figure 2D).

### Most immune cells were negatively correlated with tumor purity

**Figure 3:**
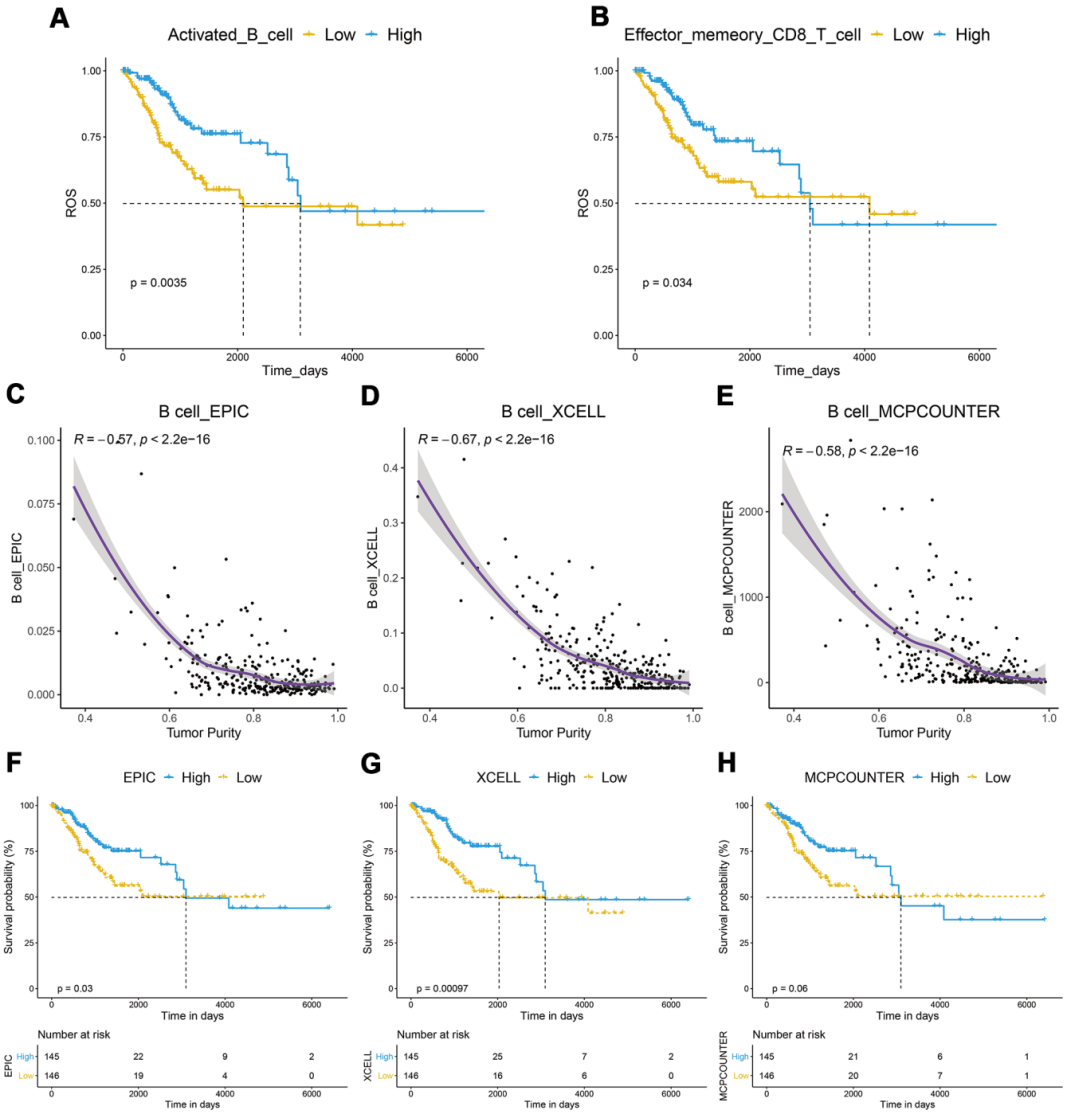
**Correlation between of immune cells with tumor purity and prognosis in CESC.** (**A**, **B**) A Kaplan–Meier analysis showed that CESC with a high infiltration level of activated B cells or effector memory CD8 T cells had a better prognosis. (**C**–**E**) B cell infiltration was significantly negatively correlated with tumor purity. (**F**–**H**) Kaplan–Meier analysis showed that patients with high B-cell infiltration levels had a better prognosis.

To explore whether the better prognosis of patients from the low tumor purity subgroup was related to immune cells in TME, we analyzed the correlation between the immune cell infiltration level and tumor purity. The results showed that the infiltration level of 19 kinds of immune cells was significantly negatively correlated with tumor purity (r < -0.5, P < 0.05, Supplementary Table 2). However, Kaplan–Meier analysis showed that only the infiltration of activated B cells and effector memory CD8 T cells was closely related to CESC prognosis (Figure 3A, 3B, Supplementary Figure 2, Supplementary Figure 3). When the B cell infiltration level was evaluated by the EPIC, EPIC, and MPCOUNTER algorithms, respectively, it showed a significantly negative correlation with tumor purity (Figure 3C–3E). Patients with high B-cell infiltration levels had a better prognosis (Figure 3F–3H). Although the XCELL algorithm demonstrated that the effect of memory CD8 T cells was also negatively correlated with tumor purity, Kaplan–Meier analysis suggested that it was not significantly associated with CESC prognosis (Supplementary Figure 1G, 1H).

### Development of a B-cell infiltration-related prognostic model

**Figure 4:**
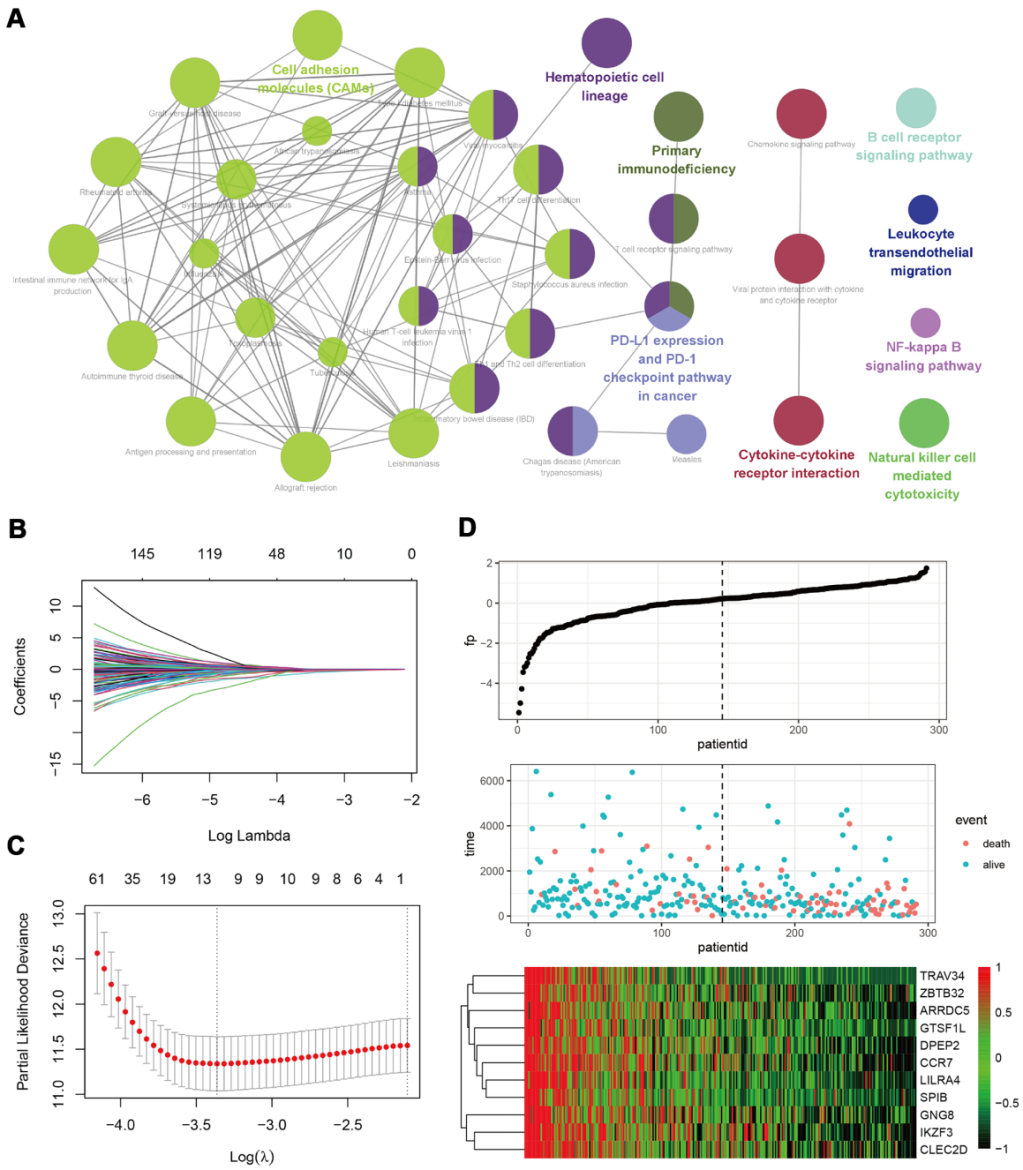
**Development of a B-cell infiltration related prognostic model.** (**A**) A KEGG enrichment analysis of 420 genes related to B-cell infiltration and prognosis. (**B**–**D**) A LASSO Cox regression model was constructed based on B-cell infiltration-related genes to calculate the tuning parameter (λ) based on the partial likelihood deviance with tenfold cross-validation. The optimal log λ value is indicated by the vertical black line in the plot.

We speculated that the B-cell infiltration level in tumors might be the key factor related to the better prognosis of CESC patients with low tumor purity. Correlation analysis was then performed to identify the genes that were strongly related to B cell infiltration in the TCGA_CESC data set. We identified 779 genes that met the p < 0.05, and r > 0.5 criteria. Univariate cox analysis showed that 420 of these genes had significant prognostic relevance (Supplementary Table 3). KEGG analysis demonstrated that these genes were mainly enriched in the cell adhesion molecules (CAMs), cytokine-cytokine receptor interaction, and hematopoietic cell lineage signaling pathways (Figure 4A). To select the key genes for a B cell infiltration prognostic model, we put the above 420 genes into a LASSO Cox regression model. We generated 11 key signature genes, namely *TRAV34*, *ZBTB32*, *ARRDC5*, *GTSF1L*, *DPEP2*, *CCR7*, *LILRA4*, *SPIB*, *GNG8*, *IKZF3*, and *CLEC2D* (Figure 4B–4D).

### Patients with low-risk scores had a better prognosis

**Figure 5:**
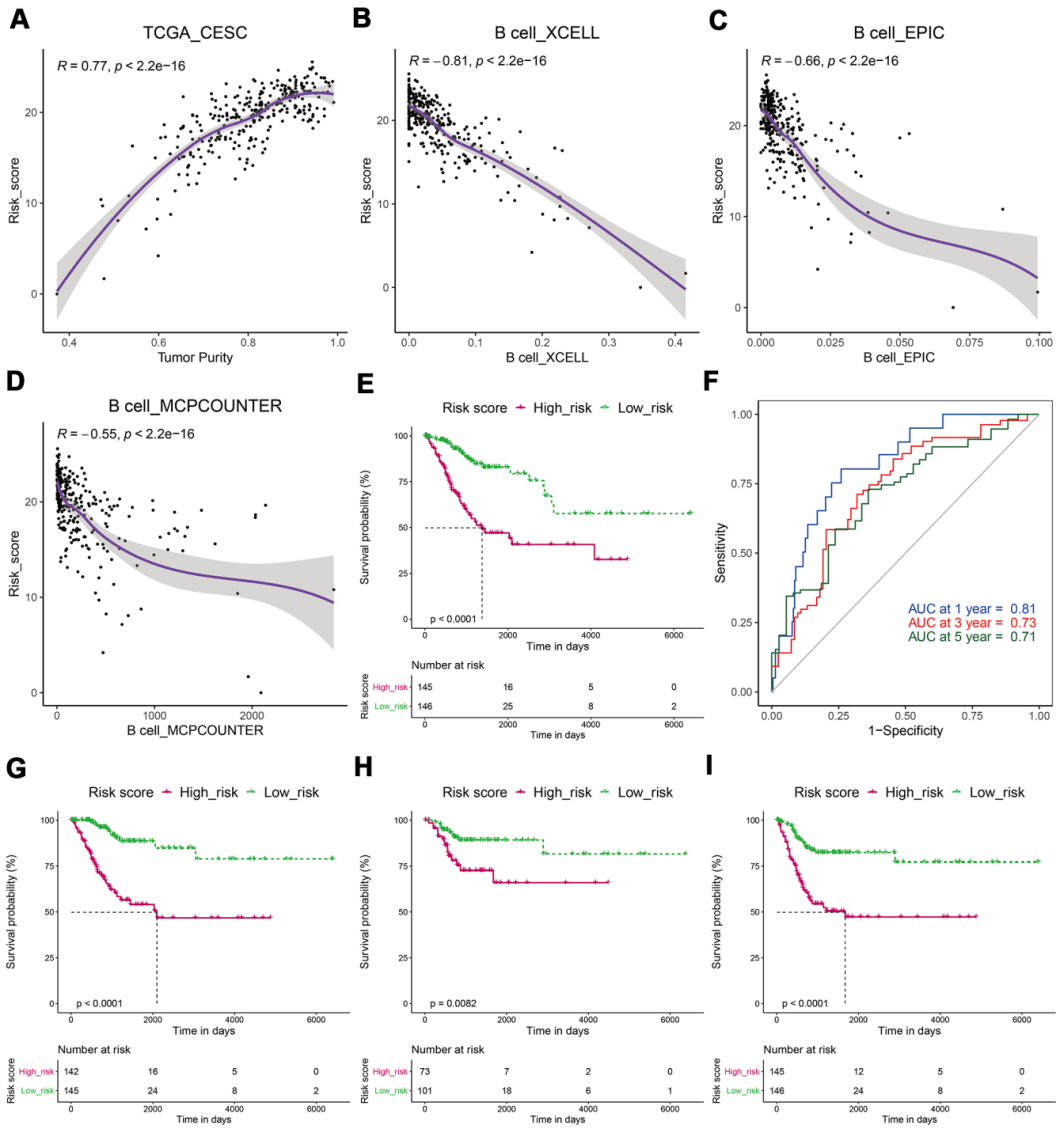
**CESCs with low-risk scores had a better prognosis.** (**A**) The risk score was significantly positively correlated with tumor purity. (**B**–**D**) The risk score was significantly negatively related to the B-cell infiltration level. (**E**) Kaplan–Meier analysis showed that patients in the low-risk subgroup had a longer OS. (**F**) ROC analysis suggested that the risk score had good predictive capability. (**G**–**I**) A Kaplan–Meier analysis showed that patients in the low-risk subgroup had longer DSS (**G**), DFI (**H**), and PFI (**I**).

We established a risk-score system related to B cell infiltration based on the expression value and corresponding correlation coefficients of the 11 key genes using the formula mentioned earlier in the Methods section. Subsequently, we analyzed the relationship between the risk score and tumor purity or B-cell infiltration level and found that the risk score was significantly positively correlated with tumor purity and negatively related to the B-cell infiltration level (Figure 5A–5D). The patients were then divided into high- and low-risk subgroups using the median value of the risk score as the cutoff value. Kaplan–Meier analysis showed that patients in the low-risk subgroup had a longer OS, and ROC analysis suggested that AUC was 0.81, 0.73, and 0.71 at one year, three years, and five years. This suggests the risk score had good predictive capability (Figure 5E, 5F). CESC in the low-risk subgroup had longer DSS, DFI, and PFI (Figure 5G–5I).

### Patients with a low-risk score had a higher immune score

**Figure 6:**
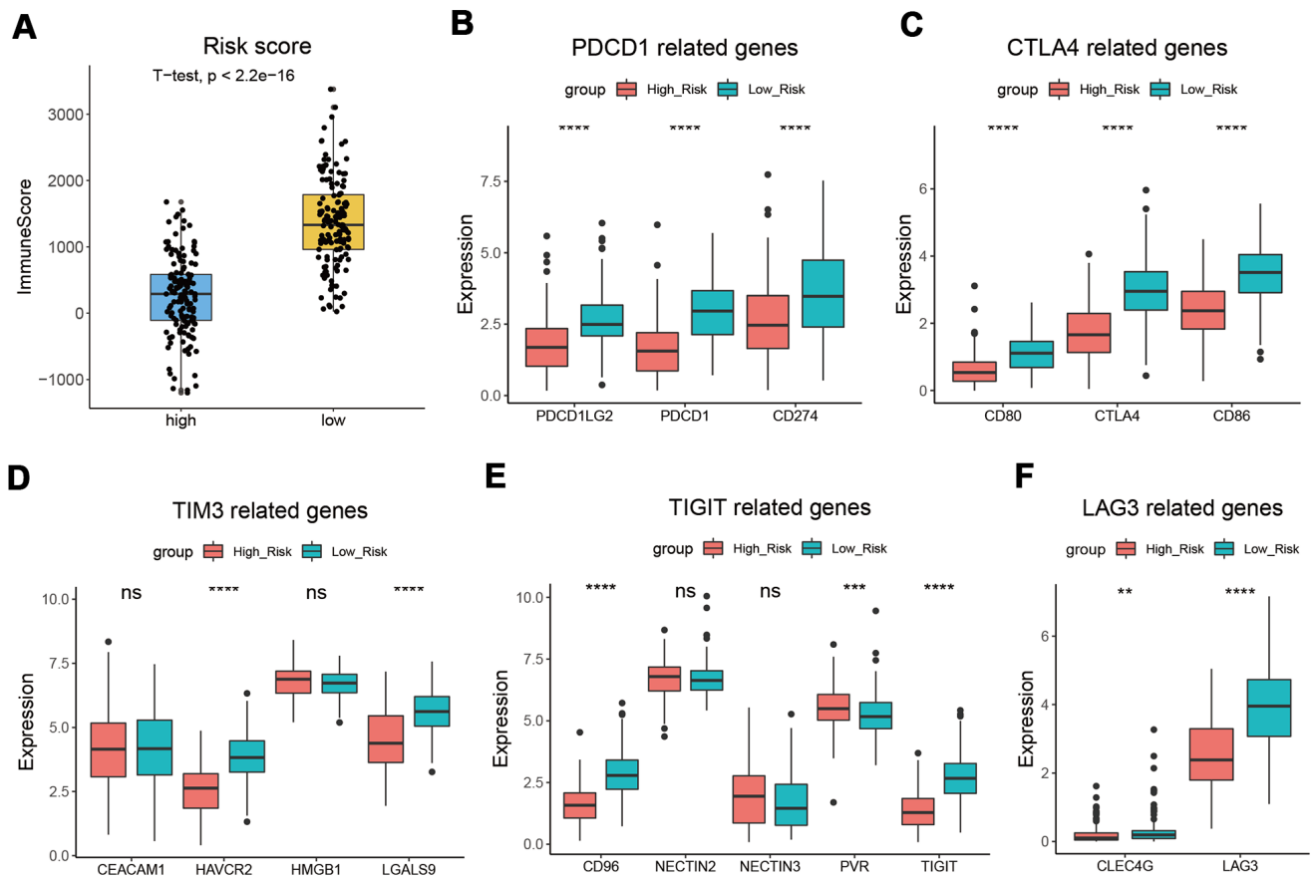
**CESC with low-risk score had a higher immune score.** (**A**) Patients from the low-risk subgroup had significantly higher immune scores than those from the high-risk subgroup. (**B**–**F**) The expression levels of PDCD1 (**B**), CTLA4 (**C**), TIM3 (**D**), TIGIT (**E**), LAG3 (**F**), and their related genes. *, *p* < 0.05; **, *p* < 0.01; ***, *p* < 0.001; ****, *p* < 0.0001.

We analyzed the correlation between the risk score and immune score and found that patients from the low-risk subgroup had significantly higher immune scores than those from the high-risk subgroup (Figure 6A). PDCD1, CTLA4, TIM3, TIGIT, and LAG3 play key roles in the immune evasion of cancer cells [20]. Subsequently, we analyzed the expression levels of PDCD1, CTLA4, TIM3, TIGIT, LAG3, and their related genes and found that they were significantly upregulated in the low-risk group (Figure 6B–6F).

### Establishment of a nomogram based on B-cell infiltration

**Figure 7:**
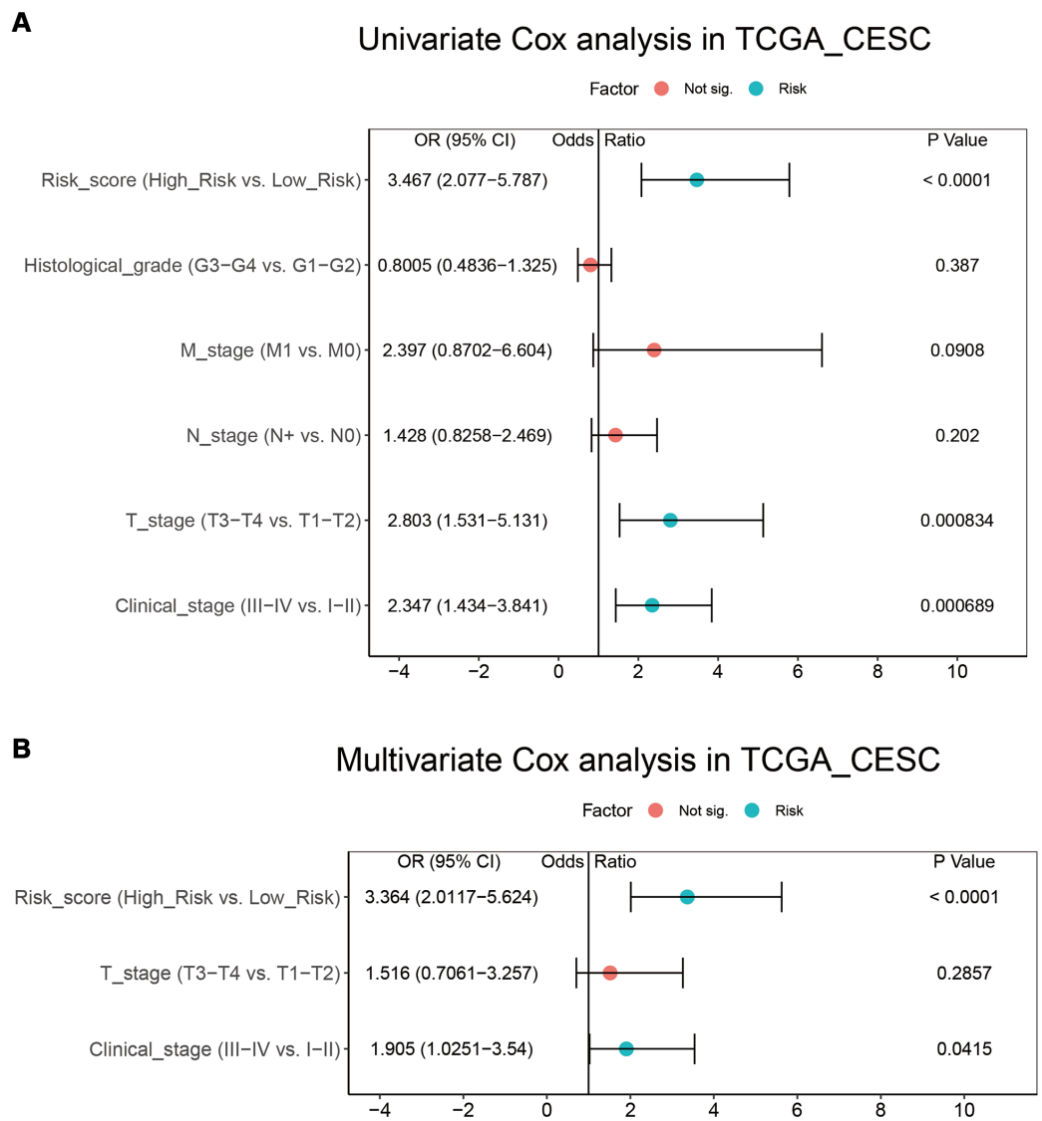
**Integration of risk score and clinical characteristics.** (**A**, **B**) Univariate and multivariate regression analysis of the relationship between risk score and clinicopathological characteristics regarding OS in the TCGA_CESC.

In the TCGA_CESC data set, we performed univariate and multivariate Cox regression analyses to assess whether risk score was an independent prognostic factor for CESC. The results of the adjustment for conventional clinical patterns, including TNM stage, histological grade, and clinical stage, indicated that risk score was an independent prognostic factor. This confirmed its robust predictive ability for the OS of patients with CESC (OR = 3.364 (2.0117–5.624), *P* < 0.0001, Figure 7A, 7B).

**Figure 8:**
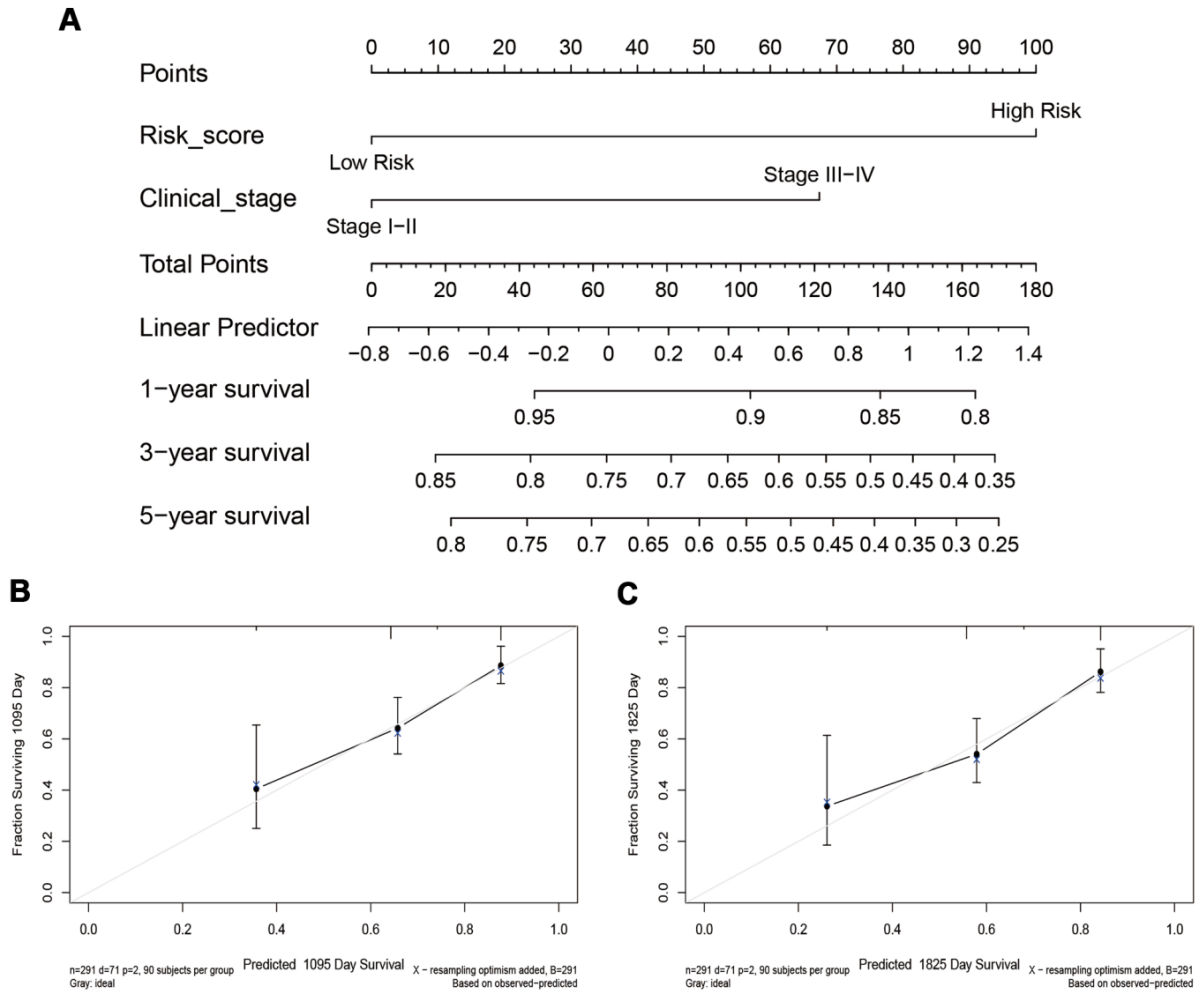
**The nomogram and calibration chart of the survival rate.** (**A**) The nomogram is based on the exhibited independent prognostic factors of CESC, including risk score and clinical stage. (**B**, **C**) The calibration chart of the 3-year and 5-year survival rates.

Subsequently, a nomogram based on the independent CESC prognostic factors, including risk score and clinical stage, was constructed and used to quantitatively assess CESC prognosis (Figure 8A). The risk score and clinical stage were assigned values according to the nomogram’s point scale. We used a horizontal line to determine each variable’s score, added the values of the two variables to get the total score of each case, and normalized this to a distribution from 0 to 180. The estimated survival rates at one, three, and five years of CESC patients were obtained by drawing a vertical line between the total point coordinate axis and each prognostic coordinate axis (Figure 8A). The calibration chart of the three and five-year survival rates suggested that the predicted results were in good agreement with the actual observations (Figure 8B, 8C). The C-index of the nomogram was 0.8055 (0.7317-0.8794), indicating it has robust predictive performance.

## DISCUSSION

Tumor tissue contains cancer cells and infiltrating and resident host cells, extracellular matrix, and secretory factors [21]. The tumor microenvironment is where tumor cells exchange substances and energy. It has an important role in tumor biology [22]. Previous studies have shown that tumor purity is associated with patient prognosis [4, 5]. According to the ESTIMATE algorithm, tumor purity was estimated based on immune score and stromal score. Tumor immune score is an important factor affecting tumor progression and immunotherapy outcomes [23]. In this study, TCGA data sets were used to calculate the tumor purity of four common gynecological cancers. We found that tumor purity was only significantly correlated with CESC prognosis (Figure 1). Interestingly, there was no significant difference in TMN stage, clinical stage, and histological grade between the high and low tumor purity subgroups in CESC (Supplementary Figure 1). This result may explain why some patients with the same TNM stage had significantly different therapy outcomes. Subsequently, we analyzed the proportion of non-tumor cells in CESC and found that the immune and stromal scores of the low tumor purity subgroup were significantly increased (Figure 2). Qi et al. found that the high immune score group had a longer OS than the low immune score group in patients with lung adenocarcinoma [19]. Wang et al. reported that the immune and stromal scores were increased significantly with increasing tumor stage [18].

GSEA results suggested that genes in the low tumor purity group were mainly enriched in immune-related pathways, such as T cell, B cell, and macrophage pathways (Figure 2). ssGSEA results showed the infiltration levels of 28 kinds of immune cells such as CD8+ T cells and MDSC were significantly increased in the low tumor purity group (Figure 2). Fang et al. found that an increase in the CD8+ T cell subset was related to a longer OS [24]. However, EPIC, XCELL, and MPCOUNTER algorithms all suggested that activated B cell levels were closely related to prognosis (Figure 3). These results suggest that the level of B cell infiltration may be a key factor in the cervical cancer prognosis. Rosamaria et al. reported that patients with lung adenocarcinoma with high levels of B cell infiltration had a better OS than those with low levels, which is consistent with our results [25].

In patients with CESC, we identified 779 B cell-related genes, 420 of which were related to CESC prognosis. KEGG pathway analysis showed that these genes were mainly enriched in CAMs, cytokine-cytokine receptor interactions, and hematopoietic cell lineage (Figure 4). CAMs play vital roles in immunity and TME, particularly integrins, which have a dominant role in the anti-tumor response [26]. To further screen out genes central to CESC prognosis, we conducted a LASSO regression analysis and identified 11 genes (Figure 4). ZBTB32 is a ZBTB transcription factor, which can regulate B cell development and function [27]. Previous studies have confirmed that ARRDC5 gene polymorphism is associated with colorectal and pancreatic cancer susceptibility [28, 29]. DPEP2 modulates macrophage inflammation [30]. CCR7 is a CC chemokine that play an important role in immune cells [31]. SPIB plays a key role in the differentiation of mature B cells into plasma and plasmacytoid dendritic cells [32]. IKZF3 was recognized as a chronic lymphocytic leukemia driver gene associated with chromatin modification [33]. CLEC2D functions as a ligand for NKRP1A, which inhibits natural killer cell cytolytic function [34]. However, the functions of TRAV34, GTSF1L, LILRA4, and GNG8 in carcinoma remain unclear. These genes were used to calculate the B-cell related risk score, which was positively correlated with tumor purity and negatively correlated with B cell infiltration. Survival analysis showed that the risk score has good CESC predictive ability (Figure 5).

We also estimated the impact of risk score on immunotherapy and found that the immune score of the low-risk group was higher. The PDCD1, CTLA4, TIM3, TIGIT, and LAG3 expression levels in the low-risk group were higher than those in the high-risk group (Figure 6). CTLA-4 can competitively bind CD80 and CD86 with CD28, inhibiting T cell proliferation and activation [35]. As an immune checkpoint, PD-1 protects the autoimmune response by inducing antigen-specific T cell apoptosis and inhibiting regulatory T cell apoptosis [36]. TIM3 can inhibit the activity of IFN-γ-producing T cells, FoxP3+ Treg cells, and innate immune cells by suppressing their responses upon interaction with their ligands [37]. TIGIT is an inhibitory receptor that can decrease T cell and natural killer cell function by interacting with CD155 expression in the antigen-presenting cell or tumor cell [38]. LAG3, produced by activated and exhausted CD4+ and CD8+ T cells, delivers inhibitory signals to regulate immune cell homeostasis and T cell activation [39]. The high expression level of these genes suggests that the low-risk group is more suitable for immunotherapy. Univariate and multivariate Cox regression analyses confirmed that risk score, T stage, and clinical stage were independent prognostic factors for CESC. These three factors were used to construct a nomogram to quantitatively assess CESC prognosis. The C-index of the model was 0.8055 (0.7317-0.8794), indicating that the model has good predictive ability.

This study suggests that tumor purity could act as a prognostic and immunotherapeutic feature in cervical cancer. However, it has some limitations. First, the sample size used in this study is small, and more clinical samples should be collected and analyzed in the future. Second, due to the small sample size (n = 55), the conclusion that USC tumor purity was not related to prognosis might be biased.

## CONCLUSION

This study was the first to reveal CESC tumor purity and to analyze the relationship between tumor purity and prognosis. We confirmed that the B cell infiltration level is significantly correlated with tumor purity and CESC prognosis. A risk score model related to B cells was constructed, and a nomogram based on this quantitatively evaluated the prognosis of CESC, which guides clinical practice.

## Supplementary Material

Supplementary Figures

Supplementary Table 1

Supplementary Table 2

Supplementary Table 3
