# Supplementary material for: Tumor purity as a prognosis and immunotherapy relevant feature in cervical cancer
Source: Aging (Albany NY). 2021 Nov 29;13(22):24768–85. doi: 10.18632/aging.203714 (PMC8660621; doi:10.18632/aging.203714)
Supplement: Supplementary Figures [file aging-13-203714-s001.pdf]

SUPPLEMENTARY FIGURES

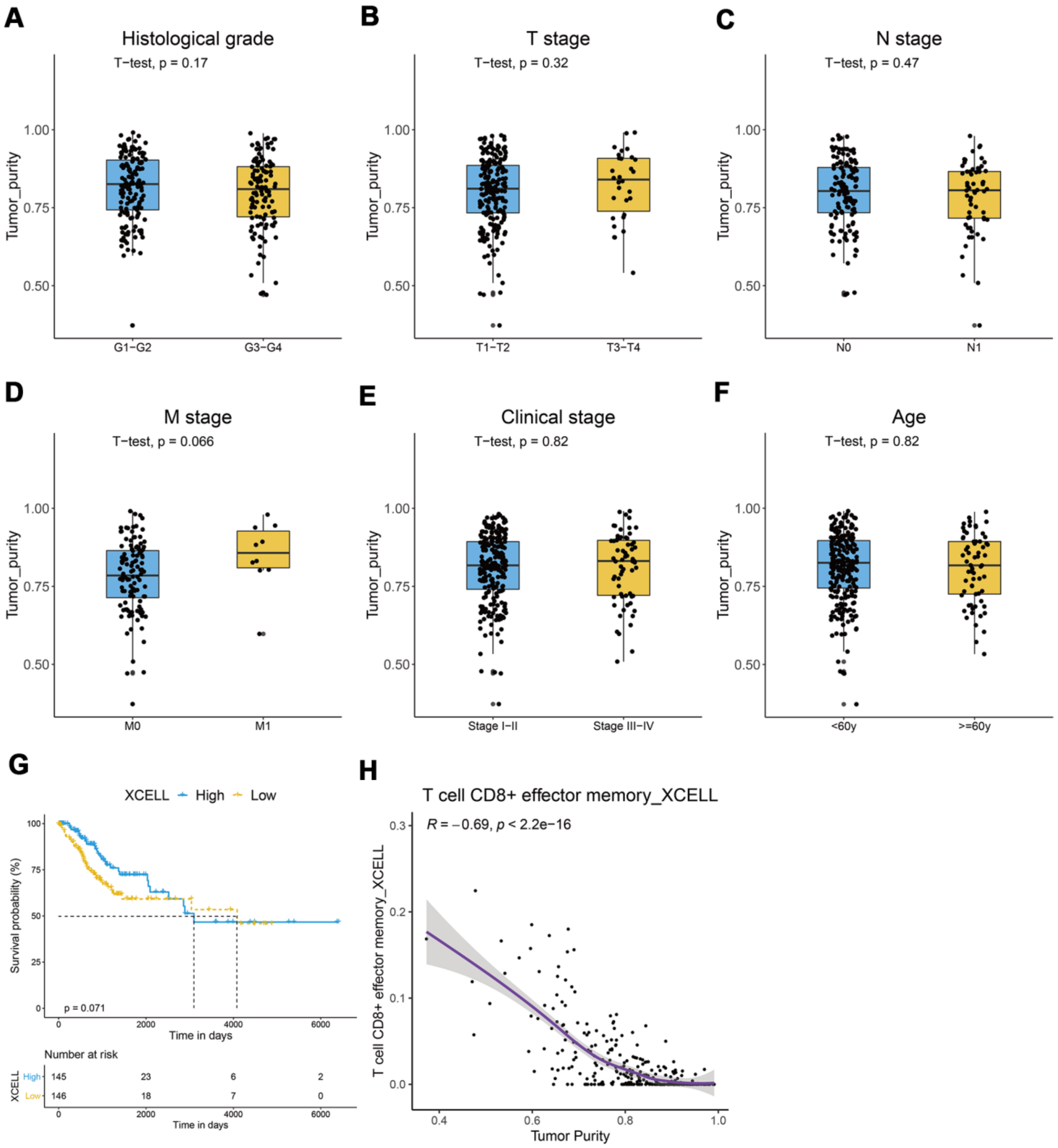

Supplementary Figure 1. Correlation between tumor purity and immunity in CESC. (**A**–**F**) Tumor purity was not significantly associated with histological grade, TNM stage, clinical stage, and age. (**G**) A Kaplan–Meier analysis suggested that the effect memory CD8 T cell was not significantly associated with CESC prognosis. (**H**) The effect memory CD8 T cell was significantly associated with tumor purity.

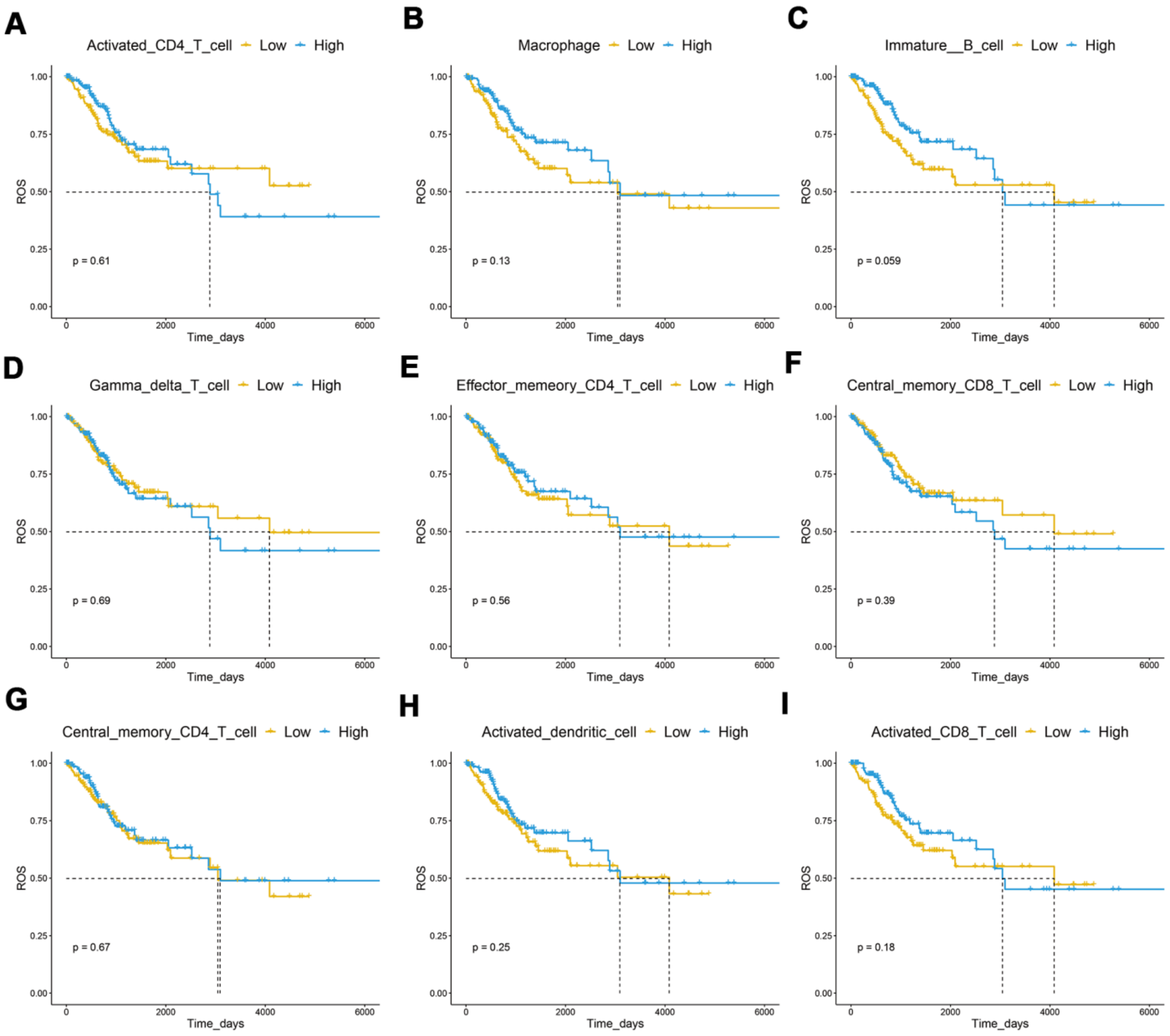

Supplementary Figure 2. The correlation between the immune cell infiltration level and OS. (**A**–**I**) A Kaplan–Meier analysis showed that activated CD4 T cell, macrophage, immature B cell, gamma delta T cell, effector memory CD4 T cell, central memory CD8 T cell, central memory CD4 T cell, activated dendritic cell, and activated CD8 T cell were not significantly associated with CESC overall survival.

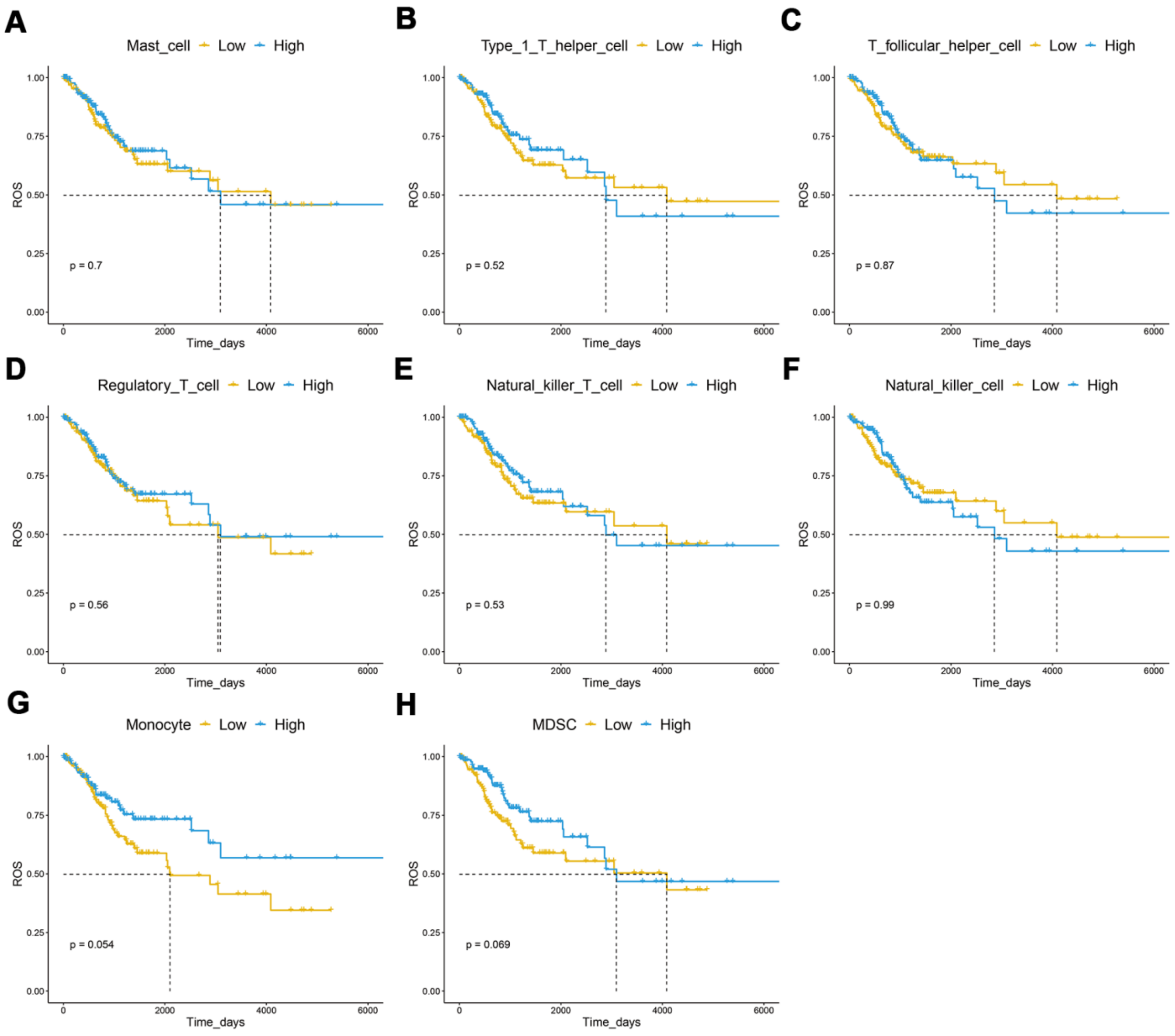

Supplementary Figure 3. The correlation between the infiltration level of immune cells and prognosis. (**A**–**H**) A Kaplan–Meier analysis showed that the mast cell, type 1 T helper cell, T follicular helper cell, regulatory T cell, natural killer T cell, natural killer cell, monocyte, and MDSC were not significantly associated with CESC overall survival.
