# Supplementary material for: Tumor purity as a prognosis and immunotherapy relevant feature in cervical cancer
Source: Aging (Albany NY). 2021 Nov 29;13(22):24768–85. doi: 10.18632/aging.203714 (PMC8660621; doi:10.18632/aging.203714)
Supplement: Supplementary Table 2 [file aging-13-203714-s003.pdf]

SUPPLEMENTARY TABLE

Supplementary Table 2. Correlation of ssGSEA data and Tumor_purity in TCGA_CESC.

Symbol	Correlation	pvalue
MDSC	-0.91283	1.71E-119
Type 1 T helper cell	-0.89922	1.92E-110
T follicular helper cell	-0.86337	1.02E-91
Immature B cell	-0.8433	1.98E-83
Regulatory T cell	-0.83903	8.13E-82
Macrophage	-0.82635	2.72E-77
Central memory CD4 T cell	-0.81962	4.89E-75
Effector memory CD8 T cell	-0.7934	4.48E-67
Activated B cell	-0.79192	1.16E-66
Activated CD8 T cell	-0.78707	2.52E-65
Activated dendritic cell	-0.73963	7.16E-54
Natural killer T cell	-0.73934	8.27E-54
Natural killer cell	-0.73424	9.97E-53
Activated CD4 T cell	-0.6845	2.35E-43
Mast cell	-0.68107	8.87E-43
Effector memory CD4 T cell	-0.6614	1.30E-39
Gamma delta T cell	-0.65421	1.63E-38
Monocyte	-0.57926	1.23E-28
Central memory CD8 T cell	-0.52831	2.98E-23
